# Understanding Usage of a Hybrid Website and Smartphone App for Weight Management: A Mixed-Methods Study

**DOI:** 10.2196/jmir.3579

**Published:** 2014-10-22

**Authors:** Leanne G Morrison, Charlie Hargood, Sharon Xiaowen Lin, Laura Dennison, Judith Joseph, Stephanie Hughes, Danius T Michaelides, Derek Johnston, Marie Johnston, Susan Michie, Paul Little, Peter WF Smith, Mark J Weal, Lucy Yardley

**Affiliations:** ^1^Centre for Applications of Health PsychologyAcademic Unit of PsychologyUniversity of SouthamptonSouthamptonUnited Kingdom; ^2^Electronics and Computer ScienceUniversity of SouthamptonSouthamptonUnited Kingdom; ^3^Southampton Statistical Sciences Research InstituteUniversity of SouthamptonSouthamptonUnited Kingdom; ^4^School of PsychologyUniversity of AberdeenAberdeenUnited Kingdom; ^5^Institute of Applied Health SciencesCollege of Life Sciences and MedicineUniversity of AberdeenAberdeenUnited Kingdom; ^6^Research Department of Clinical, Educational and Health PsychologyUniversity College LondonLondonUnited Kingdom; ^7^Primary Care and Population SciencesUniversity of SouthamptonSouthamptonUnited Kingdom

**Keywords:** qualitative research, weight loss, behavioral research, mobile apps, Internet, health, program acceptability, behavior, mixed-methods

## Abstract

**Background:**

Advancements in mobile phone technology offer huge potential for enhancing the timely delivery of health behavior change interventions. The development of smartphone-based health interventions (apps) is a rapidly growing field of research, yet there have been few longitudinal examinations of how people experience and use these apps within their day-to-day routines, particularly within the context of a hybrid Web- and app-based intervention.

**Objective:**

This study used an in-depth mixed-methods design to examine individual variation in (1) impact on self-reported goal engagement (ie, motivation, self-efficacy, awareness, effort, achievement) of access to a weight management app (POWeR Tracker) when provided alongside a Web-based weight management intervention (POWeR) and (2) usage and views of POWeR Tracker.

**Methods:**

Thirteen adults were provided access to POWeR and were monitored over a 4-week period. Access to POWeR Tracker was provided in 2 alternate weeks (ie, weeks 1 and 3 or weeks 2 and 4). Participants’ goal engagement was measured daily via self-report. Mixed effects models were used to examine change in goal engagement between the weeks when POWeR Tracker was and was not available and whether the extent of change in goal engagement varied between individual participants. Usage of POWeR and POWeR Tracker was automatically recorded for each participant. Telephone interviews were conducted and analyzed using inductive thematic analysis to further explore participants’ experiences using POWeR and POWeR Tracker.

**Results:**

Access to POWeR Tracker was associated with a significant increase in participants’ awareness of their eating (β_1_=0.31, *P*=.04) and physical activity goals (β_1_=0.28, *P*=.03). The level of increase varied between individual participants. Usage data showed that participants used the POWeR website for similar amounts of time during the weeks when POWeR Tracker was (mean 29 minutes, SD 31 minutes) and was not available (mean 27 minutes, SD 33 minutes). POWeR Tracker was mostly accessed in short bursts (mean 3 minutes, SD 2 minutes) during convenient moments or moments when participants deemed the intervention content most relevant. The qualitative data indicated that nearly all participants agreed that it was more convenient to access information on-the-go via their mobiles compared to a computer. However, participants varied in their views and usage of the Web- versus app-based components and the informational versus tracking tools provided by POWeR Tracker.

**Conclusions:**

This study provides evidence that smartphones have the potential to improve individuals’ engagement with their health-related goals when used as a supplement to an existing online intervention. The perceived convenience of mobile access to information does not appear to deter use of Web-based interventions or strengthen the impact of app access on goal engagement. A mixed-methods design enabled exploration of individual variation in daily usage of the app-based tools.

##  Introduction

### Background

Over the past decade, there has been a proliferation of digital technologies to deliver interventions designed to support health behavior change, including computer- and Internet-based platforms [1-4], social media and online social networks [5,6], and mobile phones and other handheld devices [7,8]. Feature-rich smartphones arguably offer unique advantages over these forms of digital delivery given their apparent ubiquity and widespread penetration within individuals’ daily lives combined with the opportunity to harness their context-aware sensing capabilities [9-11]. Smartphone apps have been used in a number of different ways to promote and support health, including (but not limited to) automated prompts and reminders, information provision, self-monitoring and tracking, remote monitoring by health professionals, and incorporation of social networks [9].

There has been a surge in the development of apps to support a diverse range of health issues, including health promotion and disease prevention (eg, nutrition and physical activity [12-16], weight management [17,18], protective sun behaviors [19], substance use [20,21]), self-management of chronic physical conditions (eg, diabetes [22-24], pain [25], asthma [26]), and self-management of mental health [27] (eg, anxiety, stress, and well-being [28-34], depression [35-39], schizophrenia [40]). Despite the proliferation of apps, research on the feasibility and effectiveness of app-based health interventions is still at a relatively early stage and, to date, has largely focused on exploring user needs, the development of content specific to a given health behavior, and/or identifying specific usability issues.

Apps can and have been designed as stand-alone health interventions. An alternative hybrid model is to provide larger packages of Web-based information and advice supplemented by on-the-go mobile-based access to specific intervention components [14,18,21,28]. There has been comparatively little in-depth examination of how individuals view, use, and react to a hybrid intervention model. For example, do individuals differ in their preference for using different digital formats (ie, Web vs app)? [41] Such insights are vital for understanding in what contexts and for whom specific forms of digital delivery may be more successful at promoting optimal usage of and adherence to health behavior interventions. For example, apps have shown particular promise in enabling prompting [15] and self-monitoring of health-related behavior [17,18,42-44] as well as increasing users’ awareness of health-related goals and behaviors [15,16,32,45,46].

There also appears to be little adequate exploration of how individuals actually use their mobile phones and integrate health apps in their day-to-day routines using longitudinal case study-based approaches [47,48]. The apparent ubiquity of mobile phones in our everyday lives suggests that apps will improve the accessibility, reach, and convenience of health interventions resulting in greater uptake, adherence, and subsequent health improvement. Mobile and app-based interventions appear to be welcomed by individuals living with chronic health conditions [22,24,49,50]. For example, diabetic adolescents suggested that a mobile app could be integrated within their daily routine to facilitate blood glucose monitoring and provide medication and appointment reminders [49]. However, apps may not always be more convenient and better suited to the delivery of self-monitoring components than Web-based interventions.

Qualitative studies have shown that while people are receptive to the possibility of using mobile health apps, these apps may be easily discarded [51], particularly if they are not designed for flexible, quick, and effort-free use [22,43,52,53], and that there are certain contexts in which app use can be perceived as inappropriate or embarrassing (eg, during working hours or eating out with peers [16,22]). There is also evidence to suggest that app-based tracking of behavior is not easily incorporated into individuals’ daily routines if unprompted [16], yet automated mobile prompts appear to be disliked by users when they are received at inappropriate moments [42] or too frequently [19]. These insights have largely been derived from participants’ hypothetical perceptions of health apps, experience with commercial apps, or brief interactions with app-based health behavior change interventions. Longitudinal research of actual use can improve our understanding of when, why, and how individuals experience and make use of health apps, or indeed their mobile phones, within their daily lives. This is needed for designing optimal user interfaces that enable quick and easy access to the core app components used on a day-to-day basis [54] as well as to inform the tailoring of automated prompts to ensure they are received at the right time with the right content.

We are aware of few studies that have combined quantitative analyses of intervention usage and change in self-reported outcomes with in-depth qualitative research to examine and compare individual differences in the impact of and experience of health apps. This study used a novel mixed-methods design to examine the impact of, usage, and views of a weight management app (POWeR Tracker) that was provided alongside a Web-based weight management intervention (POWeR). Facilitating users to set personal healthy eating and physical activity goals is a key behavior change technique (BCT) incorporated in POWeR. The Health Action Process Approach argues that behavior change (in this case pursuit of weight management goals) occurs through 2 phases: motivational and volitional [55]. Coping self-efficacy (the extent to which an individual feels confident in overcoming barriers to goal pursuit) and action control (awareness of intended behaviors and self-regulatory effort) are identified as key predictors of behavior [56]. This study first examined whether providing access to POWeR Tracker enhanced participants’ self-reported goal engagement and, if so, whether/how the extent of enhancement varied between participants. Goal engagement was conceptualized in this study as motivation for goal pursuit, coping self-efficacy, action control (awareness of goals, effort toward goals), and achievement of goals. Variation in each participant’s views of POWeR Tracker and their usage of it on a day-to-day basis were then examined to further understand how and why it was used and to offer explanations for any individual variation observed in the impact of POWeR Tracker access on levels of goal engagement. The efficacy of the Web-based POWeR intervention and participants’ views of the specific content provided by the Web-based intervention have been the focus of previous qualitative studies [57] and randomized controlled trials within health care [58] and community settings [59].

### The Interventions

#### POWeR

Positive Online Weight Reduction (POWeR) is a Web-based weight management intervention that was developed using the LifeGuide authoring software [60]. POWeR offers a flexible, nonprescriptive approach to weight management to foster autonomy and support users to adopt healthy behaviors that will empower them to maintain long-term weight management. POWeR is delivered over 12 sessions that become available to users on a weekly basis. Each session is comprised of a range of “POWeR tools” that support the development of self-regulatory skills, “POWeR stories” that model successful weight management strategies, and links to further sources of information and advice. A detailed overview of the iterative development and qualitative piloting of the Web-based POWeR intervention is provided elsewhere [57].

The first 3 sessions are designated as core sessions. Session 1 introduces the POWeR approach, guides users to select goals consistent with either a low-calorie or a low-carbohydrate eating plan, and review their personal motivations for losing weight. Session 2 provides tips on getting support for weight management. Session 3 guides users to select goals consistent with either a walking or mixed physical activity plan. Setting personalized goals (and developing detailed plans of how to meet those goals), reviewing goal progress, and getting Web-based feedback on goal achievement is an essential element of the POWeR intervention. From session 2 onward, participants are required to log their weight and review their goals before accessing new session content. During sessions 4-11, users are invited to choose topics of interest (eg, controlling cravings, dealing with slip-ups, eating out) after completing their weight and goal review. The final session of POWeR provides information and advice on how users can maintain their weight management over the longer term.

#### POWeR Tracker

**Figure 1:**
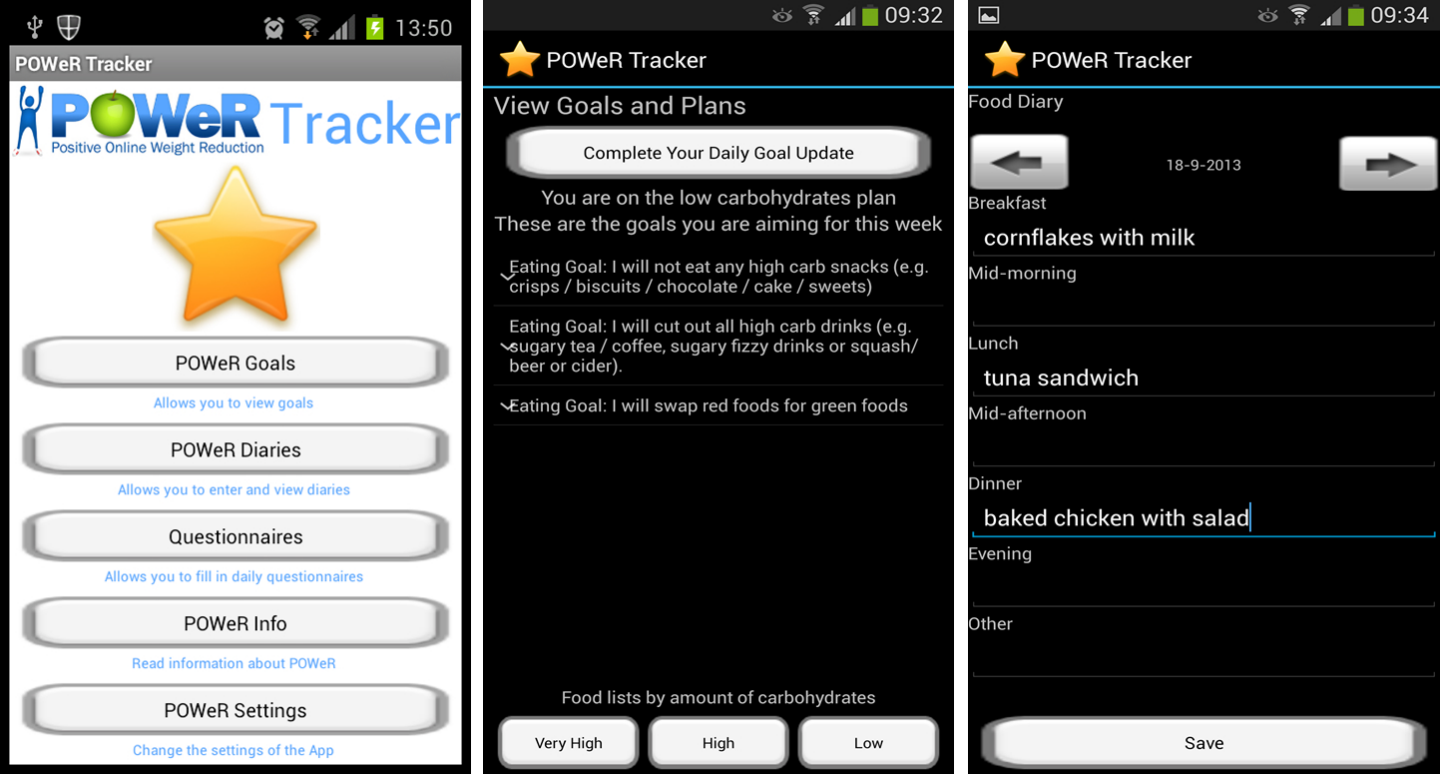
Screenshots of POWeR Tracker mobile app (left to right: menu, daily goal update, and food diary).

POWeR Tracker is an Android mobile phone app that offers users of POWeR the opportunity to keep track of their personal POWeR goals via their mobile phone (refer to Multimedia Appendix 1 for a detailed overview). POWeR Tracker is supplemental to, rather than a duplication of, the Web-based POWeR intervention. It provides a range of tools that are designed to enhance users’ awareness of and motivation to work toward their personal POWeR goals between the Web-based weekly POWeR sessions (see Figure 1). POWeR Tracker provides 2 types of tools: informational and self-monitoring. Informational tools include options to view one’s goals and plans and access selected content that was introduced during the first Web-based POWeR session, such as food lists of items that are high or low in calories or carbohydrates, one’s personal reasons to lose weight card, and advice on how keeping diaries can support weight management. Self-monitoring tools include options to receive personalized feedback on progress toward goals via completion of a daily goal update and to complete food and physical activity diaries. POWeR Tracker also offers users the ability to set up automated notifications that provide reminders to view goals or complete a daily goal update at a time of their choosing. Participants are free to cancel notifications at any time.

## Methods

### Recruitment

A volunteer sample of 13 participants was recruited using paper-based advertisements placed around the campus of the University of Southampton, UK. Eligible participants were required to have a body mass index (BMI) of at least 23, own an Android mobile phone, and have no pre-existing health conditions that would impede modification of nutrition or physical activity. Recruitment ceased when no substantially different insights of participants’ experiences of POWeR Tracker were derived (ie, when saturation was achieved).

### Design and Procedure

**Figure 2:**
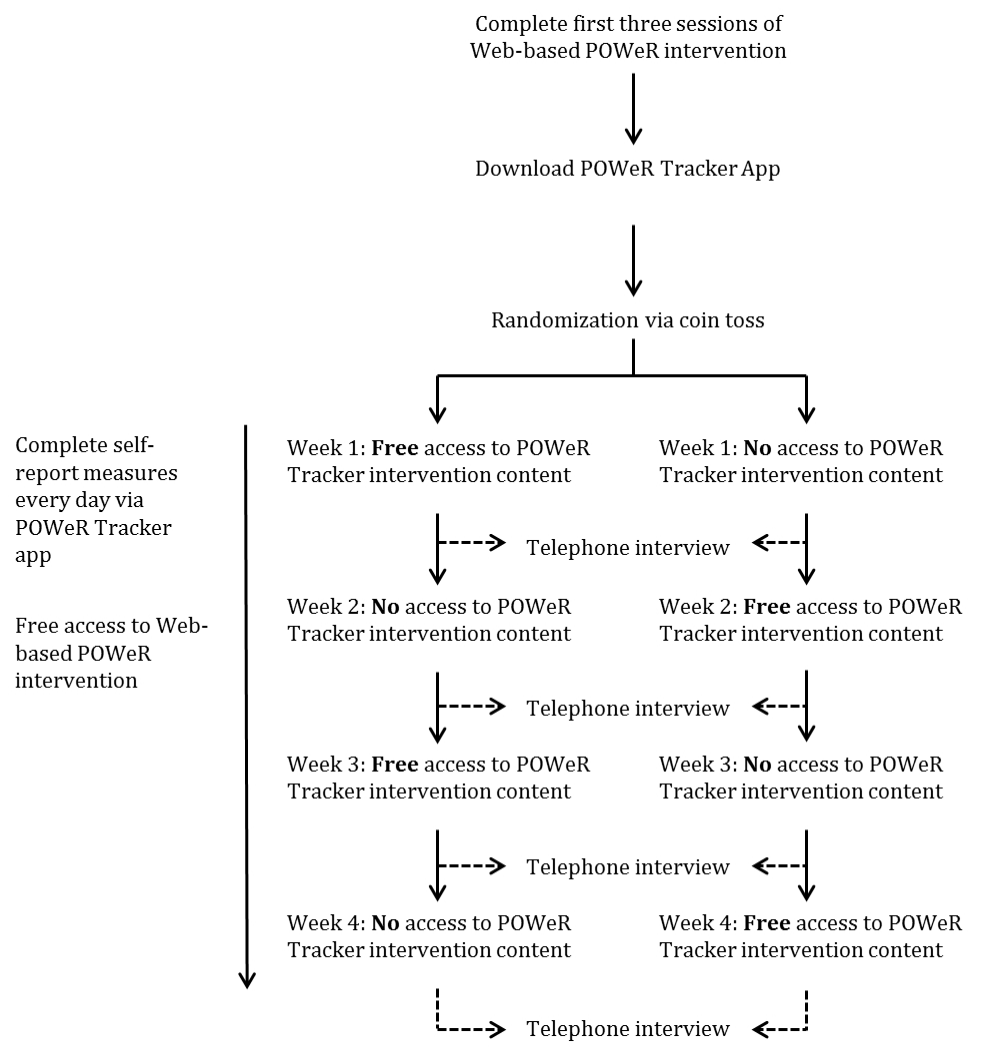
Flowchart of study design.

Data collection took place between August 2012 and August 2013 and was approved by the University of Southampton ethics committee and research governance office. Each participant was required to select their personal eating and physical activity plans and goals during the first 3 Web-based sessions of the POWeR intervention. After completing the first 3 Web-based sessions, each participant was invited to download the POWeR Tracker app and was then monitored over a 4-week period (see Figure 2). During this period, participants could continue to freely use the Web-based POWeR intervention.

When referring to the POWeR Tracker app, the phrase “intervention content” refers to the information, advice, and tools provided, whereas the phrase “daily questionnaires” refers to the self-report study measures. Access to the intervention content provided by the POWeR Tracker app was restricted and alternated on a weekly basis for each participant. Participants could either access the intervention content in weeks 1 and 3 or in weeks 2 and 4. The order of first access to the POWeR Tracker intervention content was counterbalanced across participants; participants were randomized via coin toss in blocks of 4 to receive first access during the first or second week of the study.

Participants were also required to complete a number of self-report measures of goal engagement (daily questionnaires) every day for all 4 weeks of the study via the POWeR Tracker app. In-line with the Health Action Process Approach [56], goal engagement was conceptualized in this study as motivation for goal pursuit, coping self-efficacy, action control (awareness, effort), and achievement of goals. To prevent backfilling, daily questionnaires could only be completed between 5 pm that day and 11 am the following morning. Semistructured telephone interviews were conducted at the end of each week to discuss each participant’s experiences of using POWeR and POWeR Tracker (see Multimedia Appendix 2 for the interview schedule). The interview schedule was initially developed collaboratively by LM, LD, and LY, but evolved over the course of the study based on responses from participants. Each interview was conducted by LM, LD, JJ, or SH and lasted between 5 and 38 minutes (mean 15 minutes). All study procedures and materials were initially pilot-tested with 1 user who was included in subsequent analyses (P1a). Participants were reimbursed for their time with either cash (£75) or research participation credits. Reimbursement was conditional on completion of the daily self-report measures and participation in the weekly telephone interviews. Reimbursement was not conditional on usage of the app- or Web-based intervention content. Participants were not provided with any training on how to register, download, or use POWeR/POWeR Tracker. However, participants were free to raise and receive advice for resolving any technical problems during the weekly telephone interviews.

### Measures

Usage of POWeR and POWeR Tracker was recorded automatically for each participant using the LifeGuide software, including when, how long, and in what order particular pages or screens were viewed. All participants were informed that their usage of POWeR and POWeR Tracker would be recorded. During the first 3 core sessions of the Web-based POWeR intervention, users were guided to create 3 eating goals and 3 physical activity goals. Self-reported goal engagement was recorded every day during the 4 week study period via the POWeR Tracker app to assess whether access to the POWeR Tracker app enhanced (1) motivation for goal pursuit (goal motivation), (2) coping self-efficacy (goal self-efficacy) (3) action control (goal awareness, goal effort), and (4) achievement of goals (goal achievement). Motivation, self-efficacy, awareness, and achievement were each measured for eating goals and physical activity goals using 3-item scales developed for this study (Cronbach alpha=.95-.99) (see Multimedia Appendix 3). Goal effort toward each of the 6 individual goals was measured using a 3-item scale developed by Louro et al [61] (Cronbach alpha=.87). All measures used a 7-point Likert scale that ranged from strongly disagree to strongly agree.

### Analysis

#### Between-Week Differences in Goal Engagement

Statistical analyses were performed using the R software environment version 3.0.2 [62]. Two mixed effects models were fitted to the combined daily self-report data from 12 participants to test whether there was a difference in the summary scores for each self-report measure (outcome variable) between the weeks when the POWeR Tracker app was and was not available (predictor variable) (Model 1) and, if so, whether the extent of those differences varied between individual participants (Model 2). Summary scores for each self-report measure were calculated by averaging the responses provided to each of the 3 items. One participant (P7a) was excluded from these analyses because he was unable to provide daily self-report data due to a technical fault with the POWeR Tracker app. Model 1 represented the experimental hypothesis specifying fixed (β_0_) and random effects (σ_ou_) for the intercept and a fixed (β_1_) intervention effect, where β_0_ denotes the average baseline value on day 1. Model 2 also allows for fixed (β_0_) and random effects (σ_ou_) for the intercept and a fixed (β_1_) intervention effect, but with an additional random effects (σ_1u_) on the intervention to allow for individual intervention effects. Model 2 was only fitted to data from the self-report measures that showed a significant intervention effect in Model 1. The fit of Model 1 versus Model 2 to the data was compared for self-report measures that showed a significant intervention effect. Likelihood ratio tests were used to select the preferred model fit for each self-report measure (ie, Model 1 vs Model 2). Model parameters were calculated for each individual participant where significant random effects of the intervention were observed. Both Model 1 and Model 2 allowed the individual level residuals to be correlated using an autoregressive process of order 1.

#### Website and App Usage Patterns

Usage of POWeR and POWeR Tracker was summarized for each participant to compare duration, frequency, and time of access to intervention content. Averages were also computed based on the combined usage data of all 13 participants to summarize how often and when the sample as a whole used POWeR and POWeR Tracker during the study period. Bivariate correlational analyses (Pearson *r*) were used where appropriate to examine whether observed patterns in participants’ usage of POWeR and POWeR Tracker were statistically significant.

Telephone interviews were audio recorded and transcribed verbatim. Transcripts for each of the 13 participants were analyzed using inductive thematic analysis [63]. In the first phase of analysis, each participant was considered individually so as to remain sensitive to the nuances of each individual’s experience of POWeR. This involved the development of initial codes to label each segment of text, which were then used to produce a set of themes that summarized the experiences for each participant. Analyzing each participant’s data separately ensured that unique or “deviant” views and/or experiences of POWeR were preserved when seeking to synthesize and compare data across the whole sample (in phase 2). This facilitated interpretation of individual variation observed for the impact of app access on goal engagement.

In the second phase, a thematic analysis of the themes generated for each participant (in phase 1) was conducted to generate a set of themes that captured the experiences of all 13 participants. This involved comparing the content of themes initially generated for each participant to merge and synthesize across participants while also preserving any differences between individuals in how they viewed and experienced POWeR. Participant identification numbers (eg, P1a, P11b) were used to protect the anonymity of participants, where “a” indicates first app access in week 1 and “b” indicates first app access in week 2.

## Results

### Overview

Presentation of the results will be provided in 4 sections. The first will describe the participant characteristics. The second will report on the significant associations between provision of the POWeR Tracker app and change in self-reported goal engagement. The final 2 sections will outline participants’ usage and views of the POWeR Tracker app and the concurrent Web- and app-based delivery of POWeR.

### Participants

A total of 13 (6 male and 7 female) healthy adults aged 18-52 years (median 27 years) participated. The BMI of participants ranged from 23.69 to 38.51 kg/m^2^ (median 26 kg/m^2^). All participants either completed or were currently studying for a degree. On average, participants reported using their mobile phone from between 1-16 hours per day (median 2 hours). Most of the participants cited more than 1 motivation for signing up for the study. The most common motivations were to lose weight and get fitter (both cited by 8/13, 62%), and to learn about a healthy lifestyle, earn money, and contribute to current research (all cited by 3/13, 23%). Only 1 participant (8%) reported that he/she was interested in trying new apps.

### Impact of POWeR Tracker App on Goal Engagement

Access to the POWeR Tracker app was associated with a significant increase in self-reported motivation, self-efficacy, awareness, and achievement of eating goals and a significant increase in self-reported awareness of physical activity goals. There were no significant differences in self-reported goal effort (for eating or physical goals) or in self-reported motivation, self-efficacy, and achievement of physical activity goals between the weeks when the POWeR Tracker app was and was not available. Table 1 presents the estimates for Model 1 along with the standard error for the estimate of β_1_ and the *P* value for the Wald test of the null hypothesis that β_1_=0.

**Table 1:** Estimates for Model 1 testing for fixed intervention effect.

Measure		β_0_^a^	β_1_			σ_ou_^c^	ρ^d^	*R* ^*2*^, %^e^	
			β_1_^b^	SE	*P*			Marginal	Conditional
**Eating goals**									
	Motivation	4.68	0.42	0.15	.01	1.41	.05	1.30	60
	Self-efficacy	4.53	0.34	0.15	.03	1.47	.11	.83	64
	Awareness	5.26	0.31	0.15	.04	1.19	.22	.96	58
	Effort, goal 1^f^	4.98	0.22	0.17	.18	1.29	.02	.38	52
	Effort, goal 2^f^	5.07	0.24	0.16	.15	1.27	-.04	.43	49
	Effort, goal 3^f^	4.84	0.10	0.24	.69	0.88	.40	.07	24
	Achievement	4.64	0.32	0.17	.06	1.38	.21	.79	60
**Physical activity goals**									
	Motivation	4.86	0.12	0.18	.52	1.40	.34	.10	58
	Self-efficacy	4.85	–0.09	0.18	.64	1.35	.16	.05	52
	Awareness	5.14	0.28	0.13	.03	1.38	.06	.71	68
	Effort, goal 1^f^	4.80	–0.02	0.30	.95	1.02	.19	.00	20
	Effort, goal 2^f^	4.66	-0.20	0.26	.45	1.42	.16	.18	38
	Effort, goal 3^f^	4.31	0.13	0.30	.67	1.42	.43	.07	35
	Achievement	4.56	0.14	0.21	.52	1.39	.32	.12	51

^a^β_0_ denotes average baseline value on day 1, where minimum possible score is 1 (strongly disagree) and maximum possible score is 7 (strongly agree).

^b^β_1_ denotes the average change in scores for all participants between the weeks when the POWeR Tracker app was and was not available.

^c^σ_ou_ denotes standard deviation of random effects for changes in average baseline value on day 1.

^d^For autocorrelation.

^e^ *R* ^*2*^ marginal denotes the proportion of total variation in each measure explained by access to POWeR Tracker; *R* ^*2*^ conditional denotes the proportion of total variation in each measure explained by access to POWeR Tracker and individual variability in self-report responses.

^f^Effort, goal 1-3 denotes each eating and physical activity goal set by participants.

The magnitude of change in self-reported awareness of eating and physical activity goals and self-reported achievement of eating goals as a result of access to the POWeR Tracker app varied significantly between participants. However, no significant variations between participants were observed for change in self-reported motivation and self-efficacy for eating goals. Table 2 presents the estimates for Model 2 along with standard error (SE) of β_1_, the *P* value for the Wald test of the null hypothesis that β_1_=0, the standard deviation of both the random intercept (σ_0u_) and random effects for the intervention (σ_1u_), and the correlation (ρ_01u_) between the 2 random effects. Likelihood ratio (LR) test statistics for comparing Model 1 and Model 2 and their respective *P* values are also displayed in Table 2.

**Table 2:** Estimates for Model 2 testing for individual intervention effects.

Measure		β_0_^a^	β_1_			σ_ou_^c^	σ_1u_	ρ_01u_	ρ	Likelihood ratio	
			β_1_^b^	SE	*P*					LR	*P*
**Eating goals**											
	Motivation	4.61	0.48	0.24	.05	1.59	0.63	–.55	.01	3.97	.14
	Self-efficacy	4.52	0.35	0.18	.05	1.49	0.32	–.16	.09	0.38	.83
	Awareness	5.13	0.45	0.35	.19	1.63	1.07	–.76	.06	20.59	<.001
	Achievement	4.55	0.42	0.32	.19	1.57	0.96	–.47	.07	10.52	.01
**Physical activity goals**											
	Awareness	5.11	0.31	0.22	.16	1.61	0.62	–.70	–.01	8.76	.01

^a^β_0_ denotes average baseline value on day 1, where minimum possible score is 1 (strongly disagree) and maximum possible score is 7 (strongly agree).

^b^β_1_ denotes change in scores between the weeks when the POWeR Tracker app was and was not available where the magnitude of change may be different for each participant.

^c^σ_ou_ denotes standard deviation from average baseline value on day 1.

^d^σ_1u_ denotes standard deviation of random effects for changes in scores between the weeks when the POWeR Tracker app was and was not available.

Participants P1a, P8b, and P11b reported the greatest improvement in awareness and achievement of goals during the weeks when access to POWeR Tracker was provided. In contrast, participants P3a, P5a, P6a, and P13b reported little or no improvement in their awareness and achievement of goals. Table 3 presents the estimated intercept (β_0i_) and intervention effects (β_1i_) for each individual participant where there was a significant random effect for the intervention.

**Table 3:** Estimated individual intercept (β_0i_)^a^ and intervention effects (β_1i_)^b^ for each participant.

Participant		Goal awareness (eating)		Goal achievement (eating)		Goal awareness (physical activity)	
		β_0i_	β_1i_	β_0i_	β_1i_	β_0i_	β_1i_
**First app access: week 1 of 4**							
	P1a	1.79	3.32	2.23	2.67	2.35	1.66
	P2a	3.57	0.48	3.65	0.28	3.27	0.52
	P3a	3.83	0.04	1.79	–0.38	4.07	0.02
	P4a	5.93	0.34	5.43	0.54	6.11	0.15
	P5a	6.05	0.04	5.11	–0.13	6.89	–0.20
	P6a	6.87	–0.03	4.98	–0.14	6.99	–0.05
**First app access: week 2 of 4**							
	P8b	3.86	0.78	3.18	1.11	4.05	0.84
	P9b	5.84	–0.13	5.60	0.19	3.54	0.18
	P10b	4.66	0.28	5.27	–0.06	5.41	0.16
	P11b	6.27	0.49	4.74	0.76	6.14	0.24
	P12b	6.21	0.14	6.04	0.25	5.78	0.33
	P13b	6.70	–0.32	6.57	0.03	6.71	–0.12

^a^β_0_ denotes baseline value on day 1, where minimum possible score is 1 (strongly disagree) and maximum possible score is 7 (strongly agree).

^b^β_1_ denotes change in scores between the weeks when the POWeR Tracker app was and was not available.

### Usage of POWeR Tracker App

On average, participants spent 29 minutes (SD 21 minutes) using the app-based intervention content during the 4 week study period. As a group, participants only accessed the app-based intervention content on an average of 9 of 14 (64%) days it was available. Participants also spent a longer total time using the app-based intervention content during the first week of access (mean 18 minutes, SD 11 minutes ) compared to the second week of access (mean 12 minutes, SD 14 minutes).

**Figure 3:**
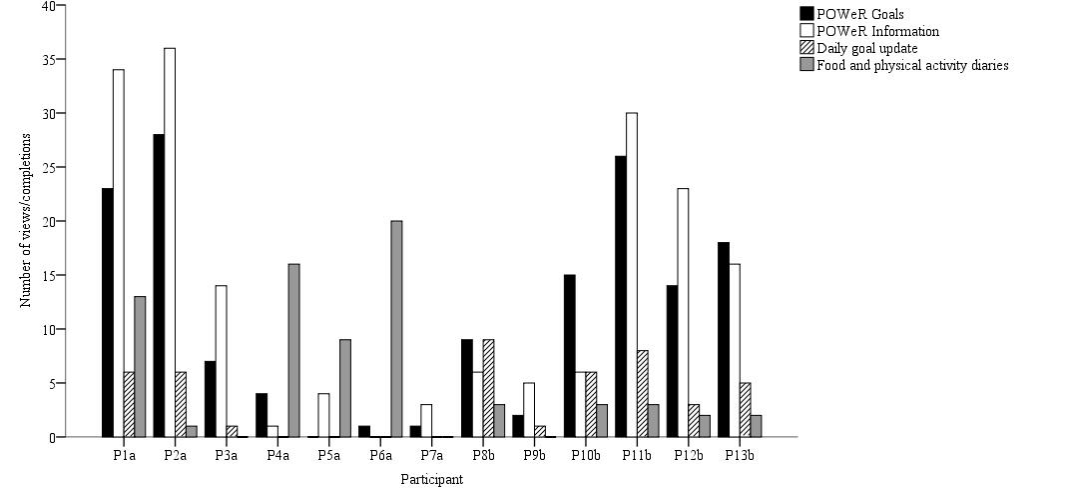
The number of times each participant viewed or completed each of the app-based intervention components.

Several individual participants did, however, access the app on most of the days it was available (see Table 4). These participants tended to request more notifications and made greater use of the app-based tracking tools, particularly the daily goal update, as compared to those participants who accessed the app on a fewer number of days (see Figure 3). A significant positive correlation was observed between total duration of app use and effect of app access on awareness of eating goals (*r*=.585, *P*=.046), achievement of eating goals (*r*=.620, *P*=.03), but not awareness of physical activity goals (*r*=.498, *P*=.10). However, more regular use the tracking-based tools was not significantly related to how strongly app access influenced goal engagement (awareness of eating goals: *r*=.525, *P*=.08; achievement of eating goals: *r*=.530, *P*=.08; awareness of physical activity goals: *r*=.387, *P*=.21).

**Table 4:** Summary of participants’ usage of the intervention content provided by the POWeR Tracker app.^a^

Participant		Duration of app use (minutes)				Days app used (0-14 days), n (%)	Notifications requested by participants, n		
		Total	First week	Second week	Per day,^b^ mean		Total	View goals	Daily goal update
**First app access: week 1 of 4**									
	P1a	64.30	20.38	43.92	5.36	12 (86)	4	2	2
	P2a	53.57	37.82	15.75	5.55	11 (79)	1	1	0
	P3a	13.20	9.40	3.80	2.20	6 (43)	0	0	0
	P4a	47.27	35.3	11.97	6.75	7 (50)	0	0	0
	P5a	15.48	9.53	5.95	2.21	7 (50)	0	0	0
	P6a	29.33	25.62	3.72	2.44	12 (86)	0	0	0
	P7a	1.32	1.32	0	0.33	4 (29)	0	0	0
**First app access: week 2 of 4**									
	P8b	24.08	17.23	6.85	2.41	10 (71)	17	8	9
	P9b	2.87	1.10	1.77	0.91	4 (29)	8	3	5
	P10b	20.15	17.32	2.83	3.36	6 (43)	0	0	0
	P11b	57.27	26.26	31.02	4.09	14 (100)	21	13	8
	P12b	17.08	14.2	28.95	1.42	12 (86)	26	14	12
	P13b	41.17	12.38	28.78	3.74	11 (79)	16	9	7

^a^Due to technical issues with the app, notifications were only intermittently received by 5 participants (P1a, P2a, P3a, P9b, and P11b). Therefore, the reported number of notifications requested may underestimate these participants’ usage of this component.

^b^Represents average duration of use only on days when the app-based intervention content was accessed.

### Perceived Advantages of App Access

Nearly all the participants (10/13, 77%) stated that it was more convenient to access the app than the website because their mobile phones were always with them and could be accessed on-the-go at any time (see Table 5).

**Table 5:** Overview of themes identified from participant interviews.

Theme	Overview
Convenience and accessibility: short bursts of on-the-go access	The app was considered a convenient and accessible means of accessing content because a phone is portable and can be used on-the-go in any location. Participants were happy to use the website and app in tandem if they were perceived to provide different value in different contexts.
Constant reminder and repetition	The app provided a constant reminder of participants’ goals and plans. This helped to maintain awareness of goals and keep them in mind. App-based reminders were typically not considered necessary on a daily or longer-term basis.
Motivational benefits of tracking	Participants reported motivational benefits from logging and tracking thoughts and behaviors via diaries, goal updates, and daily questionnaires. Use of tracking tools facilitated recognition of both progress and areas for future improvement.
Time-relevant use guided by lifestyle and routine	Usage of the app was tied to personal lifestyle and fluctuations in daily routine. Participants typically reported using the app during free moments or specific times when the app-based intervention content was most relevant, such as mealtimes.

The perceived convenience of the app appeared to resolve barriers to accessing Web-based intervention content; the app could be used during a spare 5 minutes while a chunk of allocated time was needed to complete a Web-based session. For example, P3a said:

Because it’s kind of in front of me all the time and in my hand, it’s easier for me if I am thinking about those kinds of issues, to look on the app rather than log on to a computer and go on the website.

An exception was P8b who reported that app-based access to intervention content was actually less convenient:

Actually I thought the website was more, for me anyway, was more accessible than the app...But that might be because in terms of my phone I kind of use my phone just for texting and calling but I’ve got my iPod for all the apps so I don’t normally connect my phone with an app...I would actually physically have to remember to use it on my phone.

Views on the convenience and accessibility of POWeR Tracker did not, however, appear to be strongly tied to observed usage or the effect of the app on goal engagement. For example, P8b used the app comparatively more and showed one of the strongest intervention effects despite perceiving app access as less convenient than Web access. P3a used the app comparatively less and showed one of the weakest intervention effects despite perceiving app access as more convenient than Web access (see Table 3).

Most participants agreed that another primary benefit of the app was that it provided a constant reminder of one’s POWeR goals (see Table 5). Figure 3 illustrates that the static information screens (ie, goal lists, food lists) were the most frequently viewed parts of the app for more than half the participants (9/13, 69%). This constant reminder of goals was reported to improve focus, awareness, and motivation. For example, P1a said:

You are always going to have goals in the back of your mind and reasons why you want to do things, but having it written down somewhere where you can just go and have a look at it is quite good. I mean that is quite a good thing to have because obviously if you write it down on a piece of paper you are most likely not to look at it well I don’t think I would to have an app to hand 24/7 is quite good.

Participants appeared to differ by how frequently they reported needing these reminders. Some participants stated that reminders of goals or food lists were not needed on a daily or continued basis particularly once the relevant information had been committed to memory, as P2a explains:

But once I’ve got the list of foods, once I know my targets and my goals, there is really no reason to log in...once it’s in my head I just don’t feel the need to look this stuff up.

Participants discussed how regular use of the tracking tools, including the daily questionnaires, facilitated critical self-reflection on progress and prompted further goal-directed behavior. P11b explained how answering questions about their goals each night on a Likert scale provided a useful gauge for realizing when it was time to set a new goal:

When you [‘ve made]...your goal a habit and then you change it [for a new goal], I think that’s the most profound thing [that’s happened to me during the study], [POWeR] gives you suggestions...[that] you should try and cut out drinking sugary tea or eating chocolate and then you try and do it and then you find, oh I can actually [did] that. And after [a goal] become[s] a habit you...try and do something else.

Participants suggested that the appeal and experience of using the app-based tracking tools could be optimized by providing effort-free data entry for diaries (eg, drop-down menus, barcode scanners) and personalized feedback. For example, P4a commented that the app could provide more feedback in-line with the weight graph provided during the weekly Web-based review:

Maybe a bit more feedback from what you said over the week or something... because it takes in all this information and then it could spit something back out.

### Usage of POWeR Website

Participants continued to use the POWeR website despite the perception that app-based access was more convenient (see Table 6). Total duration of app use was significantly correlated with total duration of website use (*r*=.77, *P*=.002). On average, participants spent approximately 56 minutes (SD 44 minutes) using the POWeR website and completed 3 of 4 available Web-based sessions during the 4-week study period. Although similar amounts of total time were spent on the website during the 2 weeks when participants did (mean 29 minutes, SD 31 minutes) and did not (mean 27 minutes, SD 33 minutes) have access to the app-based intervention content, time spent on the website during nonapp weeks was significantly correlated with effect of app access on awareness of eating goals (*r*=.930, *P*<.001), achievement of eating goals (*r*=.849, *P*<.001), and awareness of physical activity goals (*r*=.867, *P*<.001).

**Table 6:** Summary of participants’ usage of the POWeR website during weeks when the app-based intervention content was and was not available.

Participant		Duration (minutes)			Session completion, n					
		All weeks	App weeks	Nonapp weeks	Weight and goal review completion			Extra topics viewed		
					All weeks	App weeks	Nonapp weeks	All weeks	App weeks	Nonapp weeks
**First app access: week 1 of 4**										
	P1a	127.54	6.06	123.15	3	0	3	2	0	2
	P2a	154.11	111.10	43.01	2	1	1	2	1	1
	P3a	54.78	15.42	39.35	4	1	3	3	1	2
	P4a	46.98	10.52	36.46	4	1	3	1	0	1
	P5a	17.53	6.84	10.69	2	2	0	0	0	0
	P6a	51.71	30.99	20.72	4	2	2	3	2	1
	P7a	9.74	3.42	6.31	2	1	1	0	0	0
**First app access: week 2 of 4**										
	P8b	67.06	25.32	41.74	3	1	2	3	1	2
	P9b	26.24	22.16	4.08	2	1	1	1	1	0
	P10b	0	0	0	0	0	0	0	0	0
	P11b	68.96	44.49	24.48	3	2	1	3	2	1
	P12b	40.60	33.21	7.39	3	2	1	3	2	1
	P13b	67.14	67.14	0	2	2	0	1	1	0

Participants P1a, P2a, and P11b expressed the most positive perceptions of the website compared to the other participants. All 3 stated that they were happy to use both the website and app to access different intervention content. Their usage patterns reflect this—they all spent an above average amount of time using both the website and app. P2a and P11b also discussed how app- versus Web-based access offered value within different contexts. For example, P11b described using the app on-the-go for quick updates, but using the website for more intensive support when he felt that progress toward his goals was waning. P2a said:

If we were comparing the website and the app, I spen[t] a lot of time across the two...that was all quite straightforward that worked well. Anything like recording the foods, the diaries, and the [physical] activit[ies]...I do prefer doing that on my computer and I’ve got time to do it. The app is...most useful [for] look[ing] up the lists of food and stuff like that.

In contrast, P9b and P10b commented that they would prefer to access the entirety of the POWeR intervention via the app. For example, P10b stated that they did not need to look at the additional content provided by the website as they were making good progress toward their POWeR goals by using the app alone. P9b discussed the value of accessing “quick little snapshots of information” via the app in comparison to the long, text-heavy, Web-based sessions:

Apps are just a lot more instant and I tend to try and do it when I’m on-the-go if I can as well. When I’m out and about, [I] try and do bits on the 3G if I can and just whenever I get a convenient moment, to be honest. You can work it around yourself instead of having to physically go to a computer to do it...I prefer to use an app than going [and] logging into the website, personally.

Again, this view is reflected in their usage patterns—both spent a below average amount of time using the website; P10b did not use the website at all, whereas P9b only used the website for a short period of time during the first 2 weeks of the study. However, a strong preference for exclusive app-based access did not appear to be associated with longer or more frequent use of the app. For example, P10b accessed the app on less than half the number of days it was available and spent an average of 3 minutes looking at the intervention content on each of those days, which is comparable to the time spent by other participants. P9b accessed the app less frequently and spent less time using the intervention content in comparison to other participants. A strong preference for exclusive app access was also not associated with stronger intervention effects. In fact, Access to POWeR Tracker appeared to have a stronger effect on goal engagement for participants who valued using both the app and the website (P1a, P2a, P11b) than for participants who only valued the app (P9b, P10b).

### When and Why Did Participants Use POWeR Tracker?

**Figure 4:**
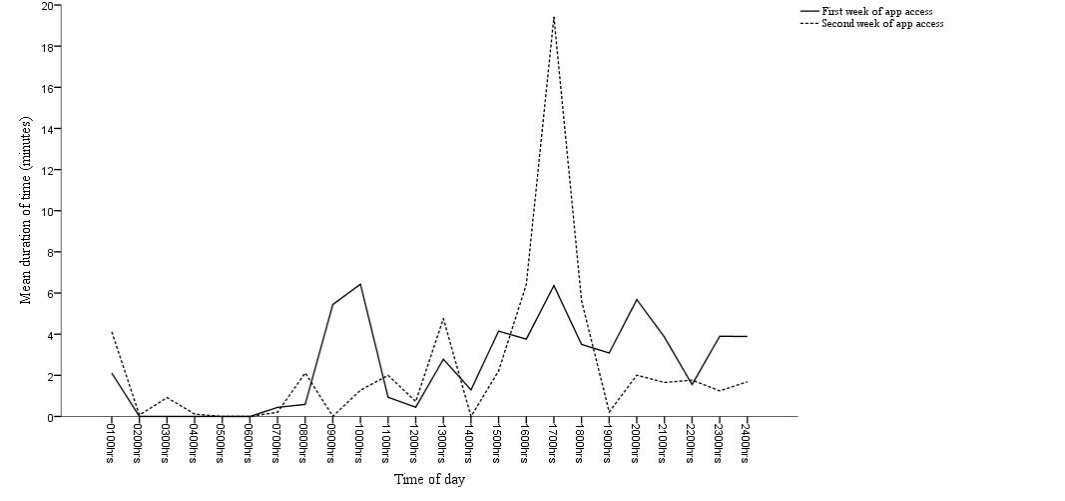
Average duration of participants’ app use (minutes) by time of day for the first and second week of app access.

Participants described using the app in short bursts during free moments of time throughout the day. On the days when the app was accessed, participants spent an average of 3 (SD 2 minutes) minutes per day using the intervention tools and 6 (SD 2 minutes) minutes per day completing the daily questionnaires. These short bursts of app use tended to be more prevalent during the morning (around 0900 and 1000 hours), lunchtime (around 1300 hours), and throughout the evening (starting from 1700 hours) (see Figure 4). For example, P11b said:

So really whenever you have free time. For me in between lectures you can go there and have a read...so that’s quite an important part for me because sometimes you have 10 minute breaks so you can just go “Oh ok, I think I will take a look at my app and see what I can do.”

There was variation between participants in when and how often the app was used within a 24-hour period. Most of the participants tended to access the app-based intervention content sporadically at different times throughout the day. For example, food lists were reportedly used at times when decisions were made about food choices, such as at the supermarket and while cooking or preparing meals. P10b described missing this point of reference when the app was not available:

When I was doing my food shopping I would usually use the card, the bit on it where it tells you which foods are good and which aren’t, I have sort of based my food shopping around those lists, but without them this week I’ve had to just try and remember what was on there. So it was like a point of reference for me.

In contrast, a few participants only accessed the app-based intervention content in the morning or the evening. For example, when asked about when she used the POWer Tracker app, P4a answered:

Usually evening, so it’s looking back on the day I mainly use it in the evening and then [I] can fill out the whole day...rather than bit by bit.

Of the 14 participants, 7 (54%) chose to set up automated notifications within the app (see Table 4). Only 2 participants discussed their reasons for not requesting notifications; one experienced a technical error preventing receipt of requested notifications (P3a) whereas the other reported that notifications were not needed because they could easily remember to use the app without them (P10b). On visual inspection, participants who requested notifications tended to be those for whom access to the app had a stronger effect on goal engagement (eg, P11b, P8b, P1a, P12b), although there were exceptions (eg, P13b). However, no significant correlation was observed between the number of notifications received and effect of app access on goal engagement (awareness of eating goals: *r*=–.07, *P*=.82; achievement of eating goals: *r*=.17, *P*=.60; awareness of physical activity goals: *r*=.11, *P*=.75).

Notifications were responded to 48 of 93 times (52%) they were received. On average, there was a 47-minute delay between notification receipt and subsequent response. View goal notifications were requested more often and prompted a greater and faster response (32/50, 64%; delay: mean 37, SD 44 minutes) than goal update notifications (16/43, 37%; delay: mean 67, SD 71 minutes). Responsiveness to notifications varied greatly both between and within individuals, ranging from the fastest response of 7 seconds and the slowest response of nearly 4 hours. The times at which notifications were requested also varied, including morning (0800 hours), lunchtime (1200 hours), early evening (1800-1900 hours), and late evening (2100-2300 hours). Participants commented that automated notifications were valuable for keeping goals in mind and prompting use of POWeR. For example, P11b said:

The thing that partly remind[ed] me to go to the app [was] the reminders because sometimes you hear your phone sound and [it] remind[s] you of something...so you tend to look at your phone so maybe if the app [could] remind me to look at my phone [more] then I think it would be a bit better...once you take a look at the [message] I’m sure people [would] spend at least a couple of minutes taking a look at the app.

P11b also suggested that notifications may have more motivational impact if they contained personalized messages related to one’s goals and plans before directing you to a particular component within the app.

Most participants explained that their app use was primarily determined by the availability of free moments within their daily routine, despite the usage prompts offered by time-relevant tools and automated notifications. Availability of free moments tended to be constrained by lifestyle factors such as work commitments and/or social commitments that were prioritized over using POWeR. For example, P3a explains that automated notifications would only evoke a response if they were delivered at the right time:

If I was to receive something like [information on exercise or healthy eating] at 11 am when I was at work I would quite likely ignore it and never go back to it again. But say it came through in the evening when I was watching TV, things might be different and I might pay more attention to it.

**Figure 5:**
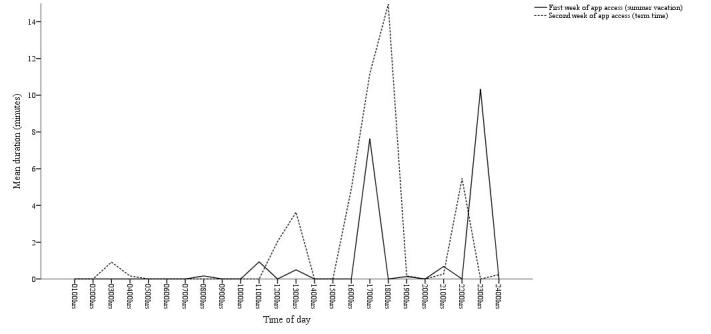
One participant’s (P1a) average use of the app (in minutes) by time of day for the first and second weeks of app access.

The timing of unavailable moments tended to be more consistent than the timing of free moments within participants’ daily routines. For example, P10b explained that she was nearly always unable to use the app during the day because she was busy with lectures, exams, and revision. However, her routine varied as to whether she had free moments during the mornings, evenings, and weekend. Access to POWeR Tracker had the strongest effect on P1a, who also discussed how app use changed after a shift in daily routine (see Figure 5). During the first 2 weeks of the study, P1a was living at home with family while on summer vacation. He described using the app fairly infrequently and typically later in the evenings because he did not have a strict daily routine and also had limited control over meal choices:

It was just over summer so I wasn’t really waking up that early. I guess if I had a 9-to-5 job then I would have used it in the mornings as well, but I wasn’t waking up till about 11 or 12 because I’m off every day, by the time I had walked the dogs and done a few other things I guess I was going on the app then.

After returning to university and living in student accommodation, P1a reported using the app earlier in the day to take advantage of free Wi-Fi on campus as well as in the early hours of the morning while out with friends. P1a also expressed a greater need for the app when living independently:

I might start using it a bit more, because when I am at home obviously you have all your comforts...like bad foods that you shouldn’t eat loads of biscuits around and stuff and so I was probably a little bit worse back home than I would be at university so I think maybe when I go shopping and stuff [now]...I’ll have a quick look at the red, green, and yellow foods and simply won’t buy the things so I don’t have the temptation.

##  Discussion

### Principal Findings

Previous research has highlighted the benefits of stand-alone app-based interventions on individuals’ awareness of their eating behaviors and physical activity levels [15,16,42,45,46]. The current study adds to this literature by demonstrating that provision of a hybrid Web- and app-based weight management intervention can encourage greater goal awareness than provision of a Web-based intervention alone. Overall, participants’ awareness of both their eating and physical activity goals was greater during the weeks when the POWeR Tracker app was available. Qualitative data indicated that this effect appeared to be stronger for participants who valued the opportunity to access both Web- and app-based content as compared to participants who preferred to use the app exclusively. Usage data indicated that this effect also appeared to be stronger for participants who spent longer using the app and longer using the website during weeks when the app was not available. Access to the POWeR Tracker app was associated with improvement in participants’ motivation, self-efficacy, and achievement of eating goals, but not physical activity goals. It is unsurprising that the POWeR Tracker app had greater impact on participants’ engagement with their eating goals than physical activity goals given that the Web-based POWeR intervention places greater emphasis on changing day-to-day dietary routines. There is also growing evidence that individuals can struggle to pursue multiple goals and plans concurrently [64].

In-line with previous research [24,50,65], the POWeR Tracker app was viewed by most participants as a convenient means of accessing desired information and tools on-the-go as needed. However, the perceived convenience of POWeR Tracker did not necessarily deter participants from continuing to use the Web-based POWeR intervention, nor was it necessarily associated with regular use of the app and stronger effects of app access on self-reported goal engagement. Participants spent similar amounts of time using the POWeR website during the weeks when the app was and was not available indicating that the POWeR Tracker was used as a supplement, rather than replacement, for the Web-based intervention. Previous examinations of hybrid interventions designed to promote self-monitoring of behavior have reported that app-based delivery was associated with better adherence to self-monitoring compared to Web-based formats [17,44]. Unlike the interventions provided by Carter et al [17] and Kirwan et al [44], self-monitoring was not the only technique promoted by the POWeR intervention. Comparison of findings from these different types of intervention suggest that apps may be associated with greater usage when they are used to facilitate one specific, repetitive daily behavior, such as diary completion or step logging.

Participants in this study varied in their use of and preference for the informational versus self-monitoring components of the POWeR Tracker app. Although most participants did use POWeR Tracker to access informational content (eg, food lists), fewer used the app to track progress toward their goals or complete food/physical activity diaries. Reasons cited for not using the tracking tools were the hassle of manual data entry and lack of personalized feedback. This contrasts with previous research revealing positive usage and views of app-based self-monitoring interventions [16,17,44], but does confirm findings from qualitative exploratory work suggesting that potential users of health apps are not always receptive to app-based self-monitoring [51,53]. As discussed earlier, self-monitoring is typically the sole behavioral target of studies that report positive usage and views of app-based delivery. It could be that these sorts of studies attract individuals who are already comfortable with self-monitoring their behavior and find it easier to integrate within their daily routines. It is also possible that the requirement for participants in the current study to complete daily measures via their mobile phones negated their perceived need for additional self-monitoring. Completion of the study measures accounted for the bulk of participants’ usage of the POWeR Tracker app, with many participants commenting that answering daily measures was a useful motivational tool.

Participants’ use of POWeR Tracker was not random and appeared to be triggered by 3 types of events: (1) relevance of app-based tools at particular times of day, (2) availability to respond or interact with the app, and (3) receipt of automated notifications. The impact of users’ availability to interact with the app and take advantage of free moments echoes reports that ability to integrate app use into one’s daily routine is an important potential facilitator and/or barrier to intervention usage [16,24,32,46]. There were no indications from participants in this study that automated reminder notifications from the app were perceived as annoying, a risk that has been highlighted previously [42]. This may be because the timing and receipt of all POWeR Tracker notifications were fully controlled by the participant, following guidelines suggested by Dennison et al [51].

### Implications and Future Research

The findings from this study offer several implications for the future delivery of app-based health interventions. First, advances in mobile technology do not necessarily render Web-based interventions redundant. Combining app- and Web-based delivery in this study improved participants’ self-reported engagement with their weight management goals suggesting that multicomponent, hybrid interventions may have the potential to enhance digital health promotion. Further research is needed to compare multicomponent, hybrid interventions to app-only interventions. This study also suggests that the benefit offered by app-based delivery in terms of convenience, does not necessarily correlate with usage or effect on outcomes, nor does it necessarily dissuade users from also engaging with Web-based interventions. That said, POWeR Tracker did not provide a complete duplication of the content and functionality offered by the Web-based intervention. Further research is necessary to examine whether the same usage patterns are observed when app-based components are equivalent to Web-based components.

Second, apps may be particularly well suited to the delivery of specific intervention components that are relevant on-the-go and that can be accessed quickly during free moments. In the current study, app-based access to select informational content (eg, food lists) was viewed as particularly useful, whereas views of app-based self-monitoring were mixed. To date, considerable attention has been paid to whether existing, often commercial, apps provide theory and evidence-based BCTs [66-71]. An additional empirical question is whether particular BCTs are more or less suited to delivery using different digital formats (eg, app vs Web). For example, can all types of behavior change tools and techniques (eg, goal setting, cognitive behavior therapy approaches) feasibly be delivered via small mobile phone screens that may typically be accessed for only minutes at a time sporadically throughout the day? Are individuals willing to use their mobile phones for extended periods of time to access detailed intervention content? Does the future of app-based intervention lie as a supplemental component that delivers only the content that is useful and convenient to access on-the-go or as stand-alone interventions, or does this depend on factors such as the target behavior or intervention complexity? These are all questions that need to be addressed by future empirical research.

Third, this study points to the value of using a mixed-methods approach to understand how individuals use and view app-based delivery of health behavior change interventions. Analysis of only the qualitative data may have overemphasized the importance of participants’ preferences for app-based delivery of intervention content. On the other hand, analysis of only the quantitative data would not have provided explanations for why the provision of a supplementary app had a greater impact for some participants compared to others. Furthermore, adopting an individualized approach enabled us to uncover differences in how participants reacted to and used the different behavior change tools provided by the POWeR Tracker app and whether these differences were related to the effect of app access on intervention outcomes. These insights will help us to improve the design of emerging app-based interventions by ensuring that they provide a range of different tools and techniques that have the potential to attract and retain a wider range of and/or higher numbers of engaged users. These insights also provide the warning that participants’ preferences for intervention delivery (ie, apps are more convenient) may not necessarily match what actually leads to better outcomes (ie, combined use of website and app).

Building a detailed understanding of when and for what reasons individuals choose to use health apps is necessary to inform the development of intelligent systems that harness the phone’s contextual sensor data to deliver real-time content or prompts at relevant and convenient moments. Adequate understanding of how and why users interact with app-based interventions on a day-to-day basis can also help to customize the design of interfaces that (1) enable users to easily access regularly used components of the app and (2) efficiently interact with components in a way that fits their actual often intermittent usage, rather than the regular usage researchers and/or programmers may intend [24,65]. Such intelligent and customized systems have the potential to both encourage and sustain usage of health-related apps.

### Limitations

A number of limitations to this study should be noted. First, this study did not use an app- or Web-only control group or a true no-intervention control group. Therefore, no definitive conclusions can be drawn regarding the comparative impact of a stand-alone app versus Web-based delivery. We also cannot rule out the role of measurement effects. As previously discussed, the daily study measures were perceived by participants as an intervention tool that offered useful motivational benefits. Findings from this study are based on a small, predominantly young, and highly educated sample limiting generalizability and statistical power. Additional studies with larger and more diverse samples are required to confirm and replicate the findings observed in this study. Ceiling effects may have limited the potential impact of access to the POWeR Tracker app on certain individual’s goal engagement, particularly goal awareness. Indeed, the participant who showed the strongest intervention effect (P1a) also had the lowest baseline values on day 1. All participants were motivated to learn about adopting a healthier lifestyle and some fell within the upper end of the healthy BMI range. It is unclear whether clinically overweight/obese individuals would interact with POWeR Tracker in the same way or whether providing access to the POWeR Tracker app would have the same level of impact on goal engagement.

It is also possible that participants’ usage of both the Web-based POWeR intervention and POWeR Tracker app was confounded by aspects of the study design. For example, usage of the app during the evening may have been encouraged by the obligation to complete the daily study measures after 5 pm. It is also possible that the financial incentives offered for participation and the perceived obligation to prepare for the weekly telephone interviews may have led some participants in this study to use the Web-based intervention and app more regularly or in different ways than they otherwise might have. However, the fact that some participants did not use the website at all and/or used the app very infrequently indicates that this was not a concern for all participants. Additionally, there were a number of technical issues with the app software that limited how well we could explore participants’ use and perceptions of the automated notifications provided by POWeR Tracker. The findings and implications regarding how participants interacted with a supplementary app-based tool provided alongside a Web-based intervention may also be specific to the domain of weight management.

Finally, this study measured the impact of providing access to POWeR Tracker on engagement with specific eating- and physical activity-related goals, but did not examine impact on dietary consumption or total physical activity level. Thus, we cannot know whether participants’ increased awareness of their eating and physical activity goals translated into healthier dietary choices or increased activity levels. It is also not clear whether improving participants’ experience of app-based interventions can actually lead to increased usage. For example, ratings of perceived usefulness and ease of use were not strongly associated with increased use of an app-based step logging tool [44].

Additional mixed-methods studies are needed to further examine both usage of and impact on behavioral change of providing supplemental app-based interventions in the longer term (ie, beyond 4 weeks). For example, does usage continue over the longer term and, if so, do and how do patterns of usage change? What factors influence long-term usage of health apps? Is there any association between a positive user experience and subsequent usage of health apps? Can exposure to health apps facilitate long-term maintenance of health behavior change?

### Conclusions

Findings from this study suggest that supplementing a Web-based weight management intervention with an app-based tool has the potential to improve individuals’ motivation for and awareness of their healthy eating and physical activity goals. Perceived convenience of mobile-based access to intervention content enabled quick access to key pieces of intervention content on-the-go at relevant and convenient moments, but did not appear to deter use of the Web-based intervention. Using mixed-methods approaches can provide complementary qualitative and quantitative insights into how users view and use app-based health behavior interventions on a day-to-day basis and what impact app-based delivery may have on health-related goals.

## Multimedia Appendix 1

Summary of content provided by the POWeR Tracker app.

## Multimedia Appendix 2

Interview schedule.

## Multimedia Appendix 3

Scale items for measures of goal awareness, achievement, self-efficacy and motivation.

## Abbreviations

BCT: behavior change technique
BMI: body mass index
POWeR: Positive Online Weight Reduction
