# Supplementary material for: Understanding Usage of a Hybrid Website and Smartphone App for Weight Management: A Mixed-Methods Study
Source: J Med Internet Res. 2014 Oct 22;16(10):e201. doi: 10.2196/jmir.3579 (PMC4259922; doi:10.2196/jmir.3579)
Supplement: Supplementary file 1 [file jmir_v16i10e201_app1.pdf]

Multimedia Appendix 1

Summary of content provided by the POWeR Tracker app.

App component	Description
POWeR Goals	Provided a list of the three eating and three physical goals that were chosen by the user during their weekly Web-based POWeR session. Users could rate the progress they had made toward each goal each day by completing the daily goal update. After the daily goal update was completed users were presented with a personalised motivational message that was tailored to their level of reported progress. Users were awarded a POWeR star for each daily goal update completed. POWeR stars were displayed on the main menu screen as a visual indication of how regularly the daily goal update was completed. Users were restricted to completing one goal update per day and could collect up to a maximum of seven stars per week.
POWeR Diaries	Provided users with the opportunity to self-monitor food intake or physical activity. The food diary comprised seven fields: breakfast, mid-morning, lunch, mid-afternoon, dinner, evening, and other. The activity diary comprised four fields: morning, lunch, afternoon, evening. Users were able to view saved entries.
POWeR Info	Provided access to select content that was introduced during the first Web-based weekly POWeR session.<br>1) The 'food lists' summarised specific food items that are low or high in calories or carbohydrates. The food lists comprised of three screens – red, amber and green. The red screen summarised foods that are very high in calories or carbohydrates – it was recommended that the user only eat these foods occasionally. The amber screen summarised foods that are high in

	calories or carbohydrates – it was recommended the user add one of these to their eating plan each day. The green screen summarised foods that are low in calories or carbohydrates – users were invited to eat these foods freely.<br>2) The ‘reasons to lose weight card’ listed the user’s personal motivations for losing weight. Users were guided to select their personal motivations during the first Web-based POWeR session.<br>3) The ‘how can keeping a diary help?’ page presented information and advice on the benefits of keeping a daily food or physical activity diary. This page also suggested a number of questions users could consider when they reviewed their diary entries e.g. “can you see where you can swap foods in your red list for foods in your green list?”
POWeR Settings	Provided users with the opportunity to set up two automated notifications at a time of their choosing:<br>1) ‘View goal’ reminder prompted users to view their personal POWeR goals.<br>2) ‘Goal update’ reminder prompted users to complete the daily goal update.<br>Users could cancel or change the arrival time of the notifications at any time.
Questionnaires	Enabled users to complete the self-report measures for the study.
