# Supplementary material for: Understanding Usage of a Hybrid Website and Smartphone App for Weight Management: A Mixed-Methods Study
Source: J Med Internet Res. 2014 Oct 22;16(10):e201. doi: 10.2196/jmir.3579 (PMC4259922; doi:10.2196/jmir.3579)
Supplement: Supplementary file 2 [file jmir_v16i10e201_app2.pdf]

Multimedia Appendix 2

Interview schedule

Opening questions and prompts

Can you tell me about your experiences of using POWeR this week?

What was it like using POWeR?

Can you tell me about whether POWeR has been helpful to you in any way?

Tell me about the things you liked about POWeR

Tell me about the things you didn't like about POWeR

Can you tell me about how you got on with using POWeR itself?

How did you feel about the information and advice POWeR gave you?

I can see that you logged onto the POWeR website [*date/time/day*]. Can you tell me more about your experiences of that?

What did you think of the goal and weight review?

You chose to look at the session on [name of session]. Can you tell me more about that?

Can you tell me more about why you decided to log onto the POWeR website at that time?

How did you feel about using the POWeR Tracker application this week? (*If applicable)*

What did you think about it?

Can you tell me about how you got on with using the app itself?

How did you feel about receiving reminders and notifications from the app?

How did you feel about the tools in the app? (*If applicable)*

Comment on use of the app e.g. "I've noticed that you haven't used [particular tool/component]. Can you tell me more about that?" Or "I've noticed that you tend to use [particular tool/component] most often, can you tell me more about that?"

Can you tell me about when you tended to use the POWeR Tracker app? (*If applicable*)

Prompt using data about daily usage, e.g. "I can see from your data log that you tended to use the application most often [time], why was that?"

How did you feel about using the POWeR website without the POWeR Tracker application? (*If applicable)*

How does the POWeR Tracker application compare to the POWeR website?

Can you tell me more about your experiences of working toward your goals over the last week?

Can you tell me about any things that you would change about POWeR?

How do you think we could improve POWeR?

Tell me about the parts of POWeR that you wouldn't change

Can you describe any parts that you liked?

Can you describe any parts that you didn't like?
