# Supplementary material for: Understanding Usage of a Hybrid Website and Smartphone App for Weight Management: A Mixed-Methods Study
Source: J Med Internet Res. 2014 Oct 22;16(10):e201. doi: 10.2196/jmir.3579 (PMC4259922; doi:10.2196/jmir.3579)
Supplement: Supplementary file 3 [file jmir_v16i10e201_app3.pdf]

Multimedia Appendix 3

Scale items for measures of goal awareness, achievement, self-efficacy and motivation.

Each scale was proceeded by the following text: “today, using POWeR helped me to …” For each item “…” represents the participant’s specific eating and physical activity goals. Each item was rated on a seven-point Likert scale ranging from 1 = strongly disagree to 7 = strongly agree.

Goal awareness:

Think about my …

Remember my …

Keep in mind my …

Goal achievement:

Follow my …

Succeed with my …

Reach my …

Goal self-efficacy

Feel more confident about achieving my … even when it was not easy.

Feel I could stick to my … even when I found them difficult.

Feel I could stay on track with my … even when I was tempted to give up.

Goal motivation

Feel motivated to keep to my …

Feel positive about my …

Feel inspired to meet my …
